# Late Glacial rapid climate change and human response in the Westernmost Mediterranean (Iberia and Morocco)

**DOI:** 10.1371/journal.pone.0225049

**Published:** 2019-12-04

**Authors:** Gerd-Christian Weniger, María de Andrés-Herrero, Viviane Bolin, Martin Kehl, Taylor Otto, Alessandro Potì, Yvonne Tafelmaier

**Affiliations:** 1 Institute of Prehistoric Archaeology, University of Cologne, Cologne, Germany; 2 Neanderthal Museum, Mettmann, Germany; 3 Institute of Geography, University of Cologne, Cologne, Germany; 4 Institute of Prehistoric Archaeology, University of Tübingen, Tübingen, Germany; Universita degli Studi di Ferrara, ITALY

## Abstract

This paper investigates the correlation between climate, environment and human land use in the Westernmost Mediterranean on both sides of the Strait of Gibraltar during the Late Glacial. Using a multi-proxy approach on a sample of 300 sites from the Solutrean and Magdalenian of the Iberian Peninsula and from the Iberomaurusian in Morocco, we find evidence for significant changes in settlement patterns and site density after the Last Glacial Maximum. In Southern Iberia, during Heinrich Stadial 1, hyperarid zones expanded drastically from the south-eastern coast to the West through the Interior. This aridification process heavily affected Magdalenian settlement in the South and caused a strong decline of hunter-gatherer population. Southern Iberia during Heinrich Stadial 1 turned out to be a high-risk environment when compared to Northern Iberia. At the same time, the Late Iberomaurusian of Morocco, although considered to be situated in a high-risk environment as well, experiences an increase of sites and expansion of settlement area.

## Introduction

Climatological research focusing on the coring of ice sheets, marine and lake sediments undertaken during the last decade has given evidence of rapid climate changes during Late Pleistocene Europe [1]. These repeated oscillations are expected to have significantly influenced the demographic development, land use and mobility of hunter-gatherer groups [2–4]. Latest advances in palaeogenetic studies of European Late Pleistocene populations indicate repeated migration events and population turnover [5,6]. These results go hand in hand with archaeological studies on Late Pleistocene palaeodemography based on settlement patterns and land use. These show clear evidence of population fluctuation in hunter-gatherer groups [3] and have stimulated the formulation of various models such as the Repeated Replacement Model (RRM) [7], which could be combined with the Adaptive Cycle Model [8,9]. Detailed analysis of archaeological site distribution and raw material procurement systems as well as land use patterns allow estimates of population density and mobility [10–13]. Therefore, a methodological toolset is available to test population change, cultural change and its relation to climate change. Crucial for this approach is a high-quality data record.

In the Westernmost Mediterranean, there is a long research history of Palaeolithic sites [14] that is accompanied by a rich palaeoclimate record from the Atlantic and the Mediterranean Sea as well as from terrestrial geo-archives [15,16]. In addition to this, the Iberian Peninsula (IP) and Morocco are known for their high bioclimatic diversity, from hyperoceanic temperate bioclimates in the Cantabrian Mountains to the hyperdesertic tropical climate at the northern fringe of the Sahara [17]. Furthermore, the Mediterranean climate today is highly seasonal in temperature and precipitation. Reconstruction of Holocene Rapid Climate Change shows evidence of a complex forcing of seasonal differences, leading to climate instability and vulnerability [18]. This combination of archaeological and palaeoclimatological data makes this area an ideal test case for the study of hunter-gatherer behaviour in relation to environmental changes.

Evidence for climate change is documented in a multitude of marine and terrestrial proxy records published during the last decades. As summarized by a series of excellent reviews [18–20], close temporal correlations between climate or vegetation changes in Iberia with stadials and interstadials recorded in the North Atlantic marine and the Greenland ice cores can be observed. The direction and magnitude of regional temperature and moisture changes in the Iberian Peninsula and the Moroccan Maghreb, however, are subject to ongoing research. For instance, palaeoclimate modelling suggests that during the Last Glacial Maximum (LGM), intensified west wind drift might have brought precipitation higher than today to the IP [21,22], but proxy records do not corroborate this [18,19]. For Heinrich Stadial 1 (HS 1, 18.0 to 15.6 ka BP acc. to Sánchez-Goñi and Harrison 2010 [16], dry and moist as well as cold and comparatively warm subphases were reconstructed for different regions in the IP [23]. It is generally accepted that HS 1 included the driest and coldest climatic conditions in the Western Mediterranean after the LGM and before temperature and precipitation rose again during Greenland Interstadial 1 (GI-1) (Bölling/Alleröd). A spatially detailed palaeoclimate modelling study for the Iberian Peninsula [24] clearly reflects this notion of dry and cold conditions during HS 1 with reduced annual rainfall and temperature, increased seasonality of rainfall and increased aridity in comparison to the LGM.

During the last years, our research group compiled a rich dataset from fieldwork at archaeological sites and at geo-archives and combined this empirical data in a meticulously controlled database of secondary archaeological data (S1 Table). In a previous study on Iberia, we were able to show that the number of sites after the Middle Palaeolithic first decreases and then gradually increases again, reaching a maximum during the LGM in the Solutrean. The south of the Iberian Peninsula in particular experiences an extraordinary increase in site numbers [3]. Here, we update the Solutrean data record and compare its extraordinary setting with the following Magdalenian, in order to study changes in human settlement pattern during the Late Glacial.

**Fig 1:**
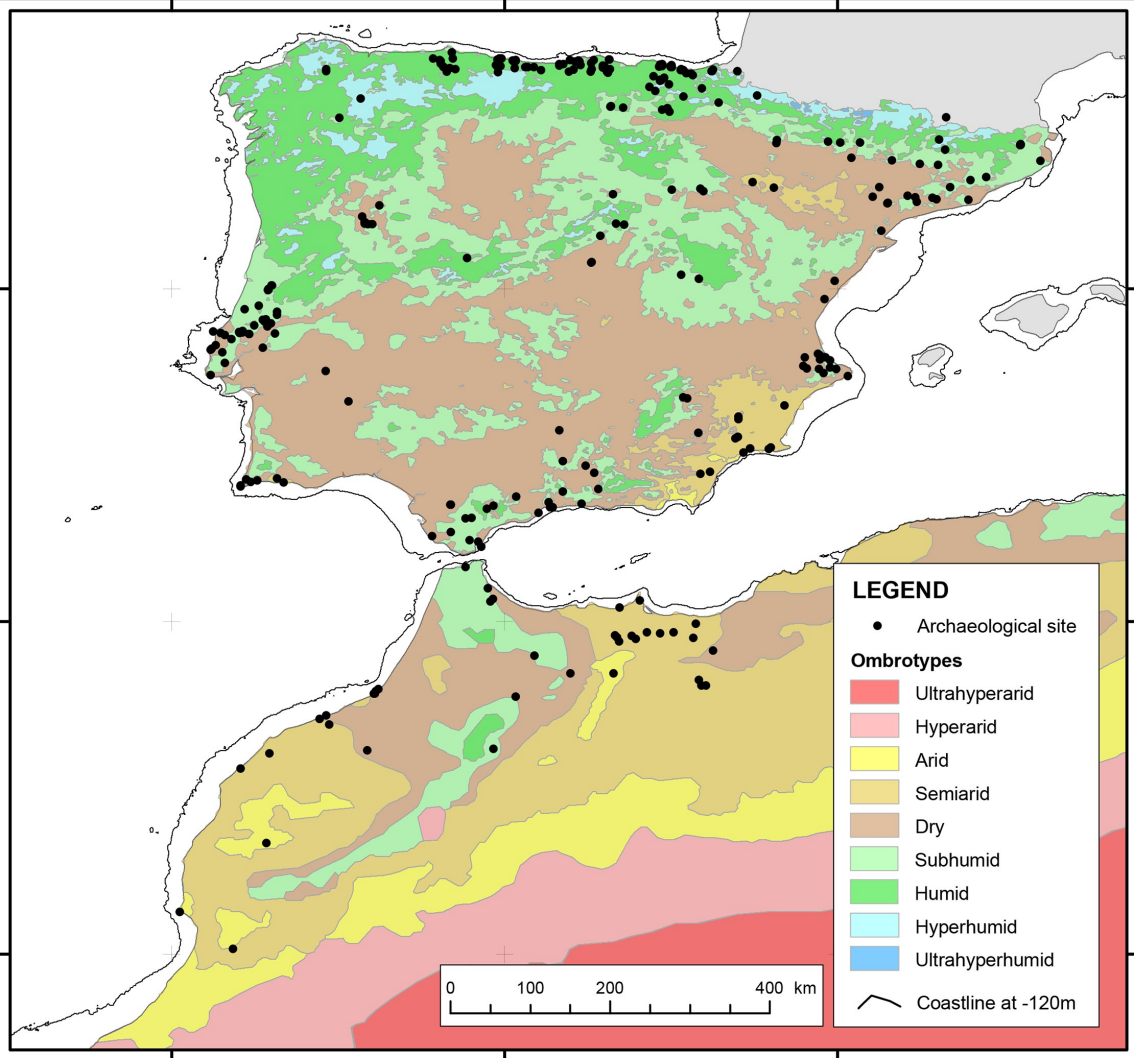
Archaeological sites with Solutrean, Magdalenian, Late or Early Iberomaurusian occupation layers compiled for this study on an ombrotype map of the Worldwide Bioclimatic Classification System (1996–2015) for Iberia (original scale 1:1 Mio.) and Africa (original scale 1:25 Mio.) [17]. For names of the sites refer to S1 Table.

Concerning Morocco, a strong decrease of site numbers after the Middle Palaeolithic resulting in a settlement gap is also evident. A very slow increase of site numbers begins only in the LGM [25]. Therefore, we analyse the distribution of Iberomaurusian sites and the resulting patterns of land use in the LGM and the Late Glacial. Our main objective is to study, for the first time, similarities and differences in human response to climate changes on a broad scale on both sides of the Strait of Gibraltar during the LGM and the Late Glacial. We assume that the location of archaeological sites in our study area (Fig 1), including caves, rock shelters and open-air sites, is not random, but reflects a preferential choice for habitats related to the presence of critical resources such as prey, water, raw material for lithic production and availability of shelter [11]. Plotted on the ombrotype map of the Worldwide Bioclimatic Classification System (1996–2015) [17], many Solutrean and Magdalenian sites on the IP are found in the modern-day humid ombrotype (Fig 1). In contrast, sites in the large and currently dry areas in the central and southern parts of Iberia are underrepresented. Next to the humid North of Iberia, the south-eastern coast of Spain and Portugal north of Lisbon evidence comparatively high site numbers. Very few sites were detected in the interior of the IP, a region probably abandoned in the early Upper Palaeolithic. Repopulation proceeded only slowly during the Late Gravettian, the Solutrean and the Magdalenian [3,26].

The site location and present-day environmental data (Fig 1) suggest that Iberia can be roughly divided along the 40° N latitude into a comparatively densely populated and moist northern half and a less densely populated and dry southern part. The significance of a split along of 40° N has proven practical in an earlier study [3] and is supported by data on Holocene climate [27]. Although other subdivisions are also possible depending on the corresponding parameters, we follow this broad subdivision to facilitate the overall comparison of Iberia and Morocco. In Morocco, fewer Iberomaurusian sites are recognized than in contemporaneous time frames on the IP. However, different climatic zones were occupied, and regional differences in site densities within the Iberomaurusian can be recognized as well.

These regional and diachronic disparities in site density could be the result of changes in habitat suitability or in patterns of land use and adaptive strategies, and therefore are the starting point of our analysis. In order to quantify diachronic and regional changes in occupation density, different methodological approaches have been applied [3,4,28,29]. We use multiple methods, most notably GIS tools, to examine these disparities. Some tools have been previously established for parts of the Iberian data record others (e.g. mating networks) are introduced for the first time. Notably, we analyse for the first time the extent and causes of changes in land use and settlement pattern of Last Glacial hunter-gatherer groups in the whole Westernmost Mediterranean on both sides of the Strait of Gibraltar.

## Materials and methods

### Archaeological records and geographical setting

The study is based on a database of archaeological sites from Iberia and Morocco dated to the interval between Heinrich Stadial 2 (HS 2) and the end of the Pleistocene (S1 and S2 Tables). The record comprises a sample of 300 sites from the Solutrean and Magdalenian of the Iberian Peninsula and from the Iberomaurusian of Morocco. The main information about the sites was derived from literature and includes the following attributes:

- geographical coordinates (recorded in WGS84)
- site type (cave, rock shelter, open air)
- archaeological composition (sites with settlement during one or multiple techno-complexes)
- stratigraphic information (single-layered or multi-layered occupation in the same techno-complex)
- radiocarbon dates

We only selected sites with reliable stratigraphies in which layers possess a clear cultural attribution given by the excavators. Additionally, we collected all reliable radiocarbon dates corresponding to these layers. Some authors assume the presence of a Badegoulian as a random diffusion from France onto the IP. The number of sites is very low and the inventories are small as well [30,31]. Due to this weak and scattered presence we assume that any influence on our data can be neglected and we exclude these very rare sites from our analysis [31]. For the Iberomaurusian, few sites without well documented stratigraphy, but with archaeological assemblages and reliable radiometric dates were kept in the database. For Morocco, the period between the end of the Middle Palaeolithic (around 30 ka calBP [32], and the onset of the Iberomaurusian is one of the most enigmatic phases—archaeological layers dated to this period are extremely rare and the classification of the lithic record is still doubtful. Considering these uncertainties, we decided not to include evidences from the so-called Early Upper Palaeolithic in the present study. Concerning the Iberomaurusian itself, current chronostratigraphic data support a division of the techno-complex into two phases, the transition corresponding to the transition from the cold-dry Greenland Stadial 2.1 (GS-2.1) including HS 1 to the warmer and moister Greenland Interstadial 1 [25,33,34]. Accordingly, in this work we define all evidences prior to the climatic amelioration of Greenland Interstadial 1 (Bölling/Alleröd) as Early Iberomaurusian, and all those in the Interstadial as Late Iberomaurusian. Sites without a clear chronological attribution were simply defined as Iberomaurusian *sensu lato* in the following analyses.

After filtering, our analyses were carried out using 143 Solutrean, 176 Magdalenian and 38 Iberomaurusian sites. The sites are grouped into three geographical areas: the North and the South of Iberia divided by 40°N and North-Western Africa between 30°N to the South and 0° to the East. Although the IP displays a climate gradient from the Mediterranean Southeast to the Atlantic Northwest and is morphologically divided into dry basins and humid mountains, which occur to the South as well as to the North of 40° N, we prefer a macro-scale subdivision of the Westernmost Mediterranean in three units: Northern Iberia, Southern Iberia and Morocco Further subdivisions are not suggested due to low sample size and lack of regionalized palaeoenvironmental proxy records. Although we expect that this division will not sufficiently reflect small-scale regional patterns, we deem our large-scale approach appropriate for the supra-regional analytical scale discussed in this paper.

**Fig 2:**
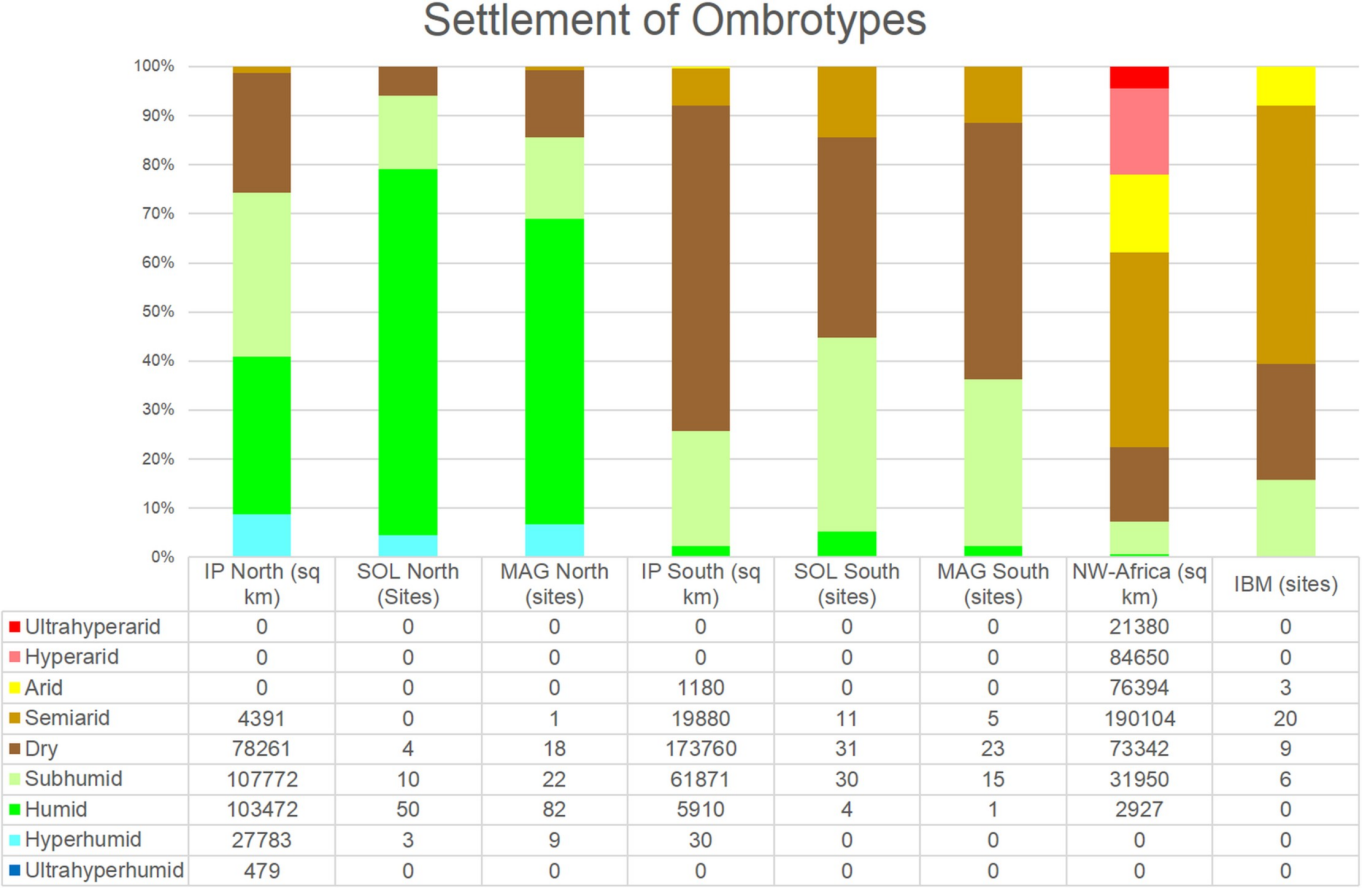
Number of Solutrean (SOL), Magdalenian (MAG) and Iberomaurusian (IBM) sites in different ombrotypes of the Worldwide Bioclimate Classification System (1996–2015) [17] in the respective study areas (IP divided by 40°N, North-West Africa between 30°N and 0°).

Our starting point is the site distribution plot on the modern ombrotype map (Fig 1), based on the observation that the annual ombrotype distribution during the LGM was not particularly different than today [24]. Moist ombrotypes in North-West Africa, Southern and Northern Iberia are overrepresented in respect to site numbers (Fig 2); this points to a preferential selection of areas which are today moister–and possibly were as well in the past–by hunter-gatherers. This preference might have been crucial during the well documented dry spells such as HS 1 within the Last Glacial in the Western Mediterranean.

The distribution of known archaeological sites may be biased by factors such as the easy recognition of diagnostic tools, research history and preferences, site preservation potential or certain geomorphological processes. Large dune fields and cover sands may have buried archaeological sites. In Iberia, large inland dune fields are known, including the Tierra de Pinares sand of the Duero Basin [35] the Manchega Plain in the Tagus Basin [36]. A compilation of the luminescence and radiocarbon data by the latter authors suggests that these aeolian sands were deposited during the Late Glacial and the Holocene (Tierra de Pinares) as well as during the LGM (Manchega plain). Near the coast, several dune fields are depicted in the Mapa Geomorfológica de España y del margen continental (Instituto Geológico y Minero, 2005) [37]. These may have been active after the Solutrean or Magdalenian occupations: luminescence dating of dune sands south of the Tagus delta yielded deposition ages between 20.5 and 11.6 ka ago, providing evidence for continuous aeolian activity in the coastal hinterland of Portugal during the last termination [38]. Other aeolian sediments include loess deposits of the Tagus Basin and at various locations in Catalonia [39,40]. These deposits can attain a thickness of over ten meters, but are a more local occurrence, probably insufficient to cause significant sampling bias. Other sites may have been lost or covered due to erosion and deposition of sediments in floodplains or on slopes. In addition, Hoyos-Gómez [41] considered the lack of Lower and Middle Solutrean layers in some sites of the Cantabrian region to be a result of reactivation of the karst. Sea level retreat during the LGM exposed large coastal plains (Fig 1) which, most probably, were inhabited by humans and flooded by rapid sea level rise during the Late Glacial. Additionally, Aura et al. [30] discuss geological and taphonomic processes that may have been responsible for mixing Solutrean with younger [42] overlying materials in few sites in Northern Iberia and the Mediterranean region.

Despite these potential biases, the compilation of sites in our database represents an exceptionally high-quality record available for analysing human occupation during the Solutrean, Magdalenian and Iberomaurusian in the Western Mediterranean. Due to this, we regard the results obtained from the following analyses as relatively robust and the data adequate to infer certain properties of prehistoric settlement and land use dynamics.

### Radiocarbon dates

Within this paper, we use 14C data and their Summed Calibrated Date Probability Distribution (SCDPDs) to recognize patterns in population dynamics (S2 Table). The approach of using "dates-as-data" is widely applied [43–48]. Criticism has been raised on interpreting the distribution and frequency of 14C dates directly as an indicator of population size. In a study on the Epigravettian in Northern Italy and South-Eastern France, Naudinot et al. [49] evidenced that variability in 14C data distribution is influenced by diachronic changes in mobility patterns. We expect similar correlations in the Western Mediterranean. Gamble et al. [50] state that climate impact concerns foremost climatic instability and population decline rather than climatic stability and population growth. The so-called cascade model [7] and additional studies in the Western Mediterranean [3] support this. However, the question of whether the emerging patterns can directly be linked to environmental change is an issue we will discuss in this paper.

A first presupposition to use dates-as-data is the validation of the radiocarbon dates. This concerns both the archaeological validity, i.e. the stratigraphic integrity, as well as the methodological validity, concerning the quality of the 14C measurement itself. Hence, our data underwent a strict quality protocol. We excluded all dates with unclear methodology, bad preservation of bone collagen, sample contamination resulting in a rejuvenated date, and dates from rock art, as these samples lack stratigraphical context. In addition, we removed all dates with a standard deviation greater than 350 years to ensure a high precision of our analysis. As previously established, single dates from Iberomaurusian sites with unclear stratigraphic sequences, but with a clear association with the cultural material were kept in the database. The exclusive use of confirmed dates should guarantee a minimum level of reliability of the emerging patterns.

Another aspect concerns the number of dates. Several authors [51,52] argue that a robust dataset comprises at least 500 dates. With an increase in sample size, the robustness of the model will increase likewise [52]. In total, 542 valid dates are available for our study area. When split into regional samples of our three macro regions, the sample size decreases to less than 500 dates; however, as radiocarbon dates are only one argument amongst many in our multi-proxy approach, and a strict quality protocol was exercised, we consider our database robust enough to generate solid patterns that can be cross-checked with additional data.

CalPal version 2016.8 [53] was used for calibration of 14C data using Intcal13 calibration curve [54], as well as for the calculation of SCDPDs. The impact of calibration on the resulting summed probability curve distribution has been repeatedly referred to [51]. In another paper, we already discussed the occurrence of calibration curve effects and their influence on summed probability plots, and it was outlined how CalPal encounters these obstacles [3]. Additionally, several studies emphasize the need for high-resolution environmental and climatic data, as their regional variability may be high. While in some cases, archaeological data constitute an integral part of climate and population simulations [50], in others, they are only used to cross-check the results [48]. Here we use two different probability plots. A coarse-grained curve assembles data from all radiocarbon dating techniques, and is refined by a second set including only AMS data.

### Site numbers, archaeological composition of sites and site distribution

Analysis of diachronic and regional change in site numbers and site density have resulted in useful characterizations of population dynamics and land use in Late Pleistocene Iberia [3,14,55] as well as elsewhere inside and outside of Europe [2,56–58]. We pursue a similar approach, using the numbers, cultural composition and distribution of sites to describe settlement patterns and compare these patterns regionally and diachronically across the Western Mediterranean.

In a previous study [3], we took the number of sites as proxy for human presence in the Middle and Early Upper Palaeolithic of Iberia, assuming that changes in site numbers and densities reflect either changes in mobility and/or population density. Site numbers per techno-complex are therefore presented here and analysed in a similar fashion. In addition to this, we utilize the archaeological composition of a site’s stratigraphy as a proxy for land use. Archaeological layers are palimpsests of human presence in a site. Sites with multiple layers represent repeated occupations over a longer period of time, while single-layered sites are interpreted as remnants of time-constrained, ephemeral human presence in the landscape. While we agree that the number of stratigraphic units is linked to settlement intensity, these sites are also part of a larger land use system. In contrast to ephemerally occupied places, the diachronic re-use of a site indicates its constant importance in the land use system and may reflect structural properties of the environment [59].

Similarly, we analyse repeated settlement of a site in broader temporal entities. If a Solutrean site is reoccupied in the Magdalenian, it may represent enduring continuity in the role that site played in the overall land use pattern. If sites are not reoccupied in the later techno-complex, it could reflect changes in resource availability (due to environmental changes or to changes in the resource selection), changes in mobility or the complete abandonment of a (micro-)region.

**Fig 3:**
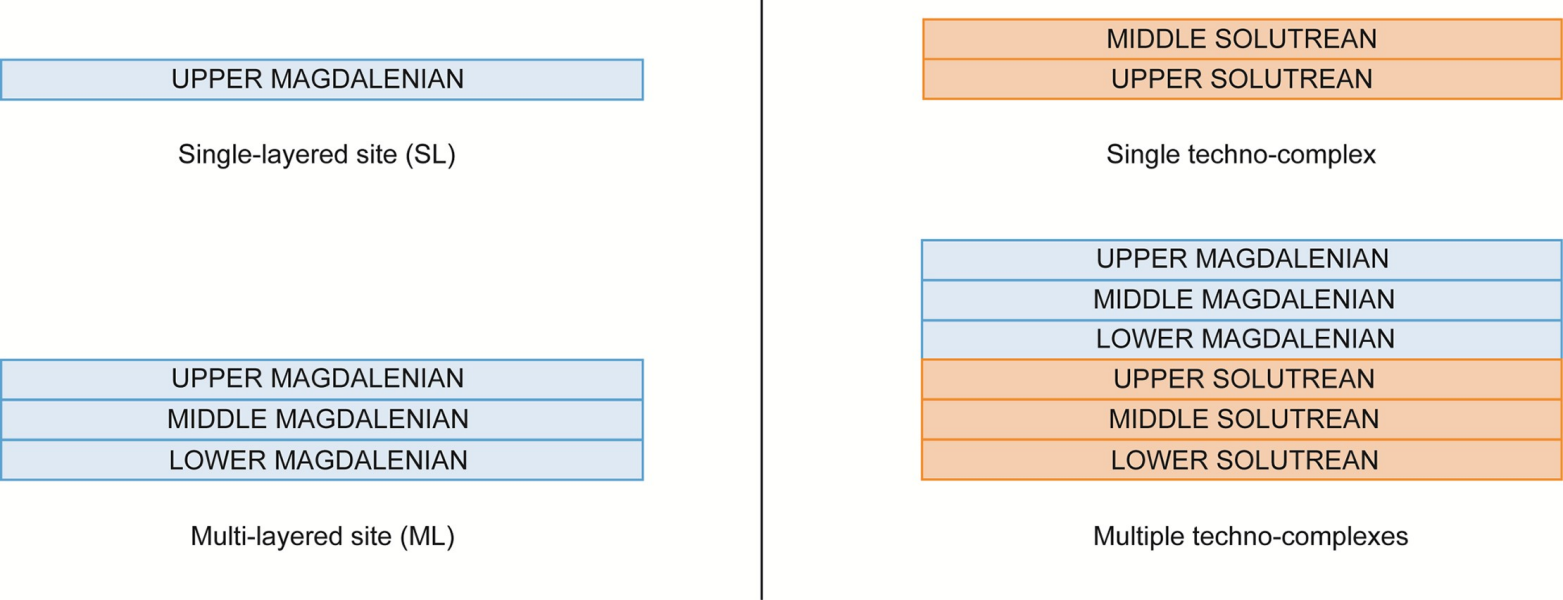
Schematic explanation of site classification: Single versus multi-layered sites and sites with one or multiple techno-complexes.

The sites in our database are therefore classified by their overall stratigraphic composition (i.e. techno-complex presence or absence) and the number of stratigraphic units per techno-complex (techno-complex sub-phases). We separate multi- or single-layered sites as well as sites with material from single or multiple techno-complexes; the schematic is presented in Fig 3. This classification is trivial for Iberia, but more difficult for Morocco. Late Iberomaurusian stratigraphies are often thick palimpsests of snail shells, making it difficult to discern clear-cut occupational episodes or geological horizons. The layer composition will therefore not be analysed directly for these sites.

Site density analyses can be applied in a similar way to the reconstruction of Palaeolithic settlement patterns. Morgan [56], Schmidt et al. [3] and Kretschmer [58] have shown how site distribution reflects settlement pattern and mobility. In addition to this, this data can be used to characterize structural properties of the environment, assuming that the location of archaeological sites is broadly related to the distribution of resources the groups utilize [11,56,60,61]. For this reason, we include Nearest Neighbour Analysis (NNA), Ripley’s K Analysis and Kernel Density Estimates into our multi-proxy approach.

The NNA analyses site density by measuring distances between sites and their closest neighbouring site [62]. Mean distances are calculated and compared to the mean distances of a random pattern spanning the same study area [60,63]. If the Nearest Neighbour Ratio is smaller than 1, the site distribution is clustered, if larger, dispersed, and statistical significance is given by the z-score and the p-value in ArcGIS 10.3.

The resulting overall density not only gives a first impression of site distribution, but likewise yields average distances between sites, which can be used to characterize and compare mobility. However, the NNA only analyses the pattern on one scale; in order to analyse site density on multiple scales, we also apply Ripley’s K analysis to our dataset [64,65]. We undertook the analysis with 100 distance bands, using identical study areas for each region (see Archaeological records and geographical setting), and plotted the difference function between the observed and expected patterns L(d)obs–L(d)exp [63,66]. Positive values indicate clustering; negative values indicate dispersal. The calculation was undertaken in ArcGIS 10.3 with a 99% confidence envelope. This method quantifiably analyses site pattern in such a way that we can compare the results between times frames in one region.

After the statistical site density analyses, we turn to the location of the sites in the landscape. Kernel Density Estimation (KDE) is a tool often used in archaeological studies to visualize and interpret site clusters [67–69]. Here, a probability function in the form of a “hill” is placed over each data point, i.e. each site, and values are summed where the individual functions overlap (see [67] for a detailed methodological description). This can be used to identify clusters [3], define cluster attribution [66], and even as a basis for population density estimates [70]. We apply the approach presented by Grove [57], who uses the KDE as a method to display human spatial behaviour.

Grove follows Isaac [71] in acknowledging that the archaeological record is comprised of merely remnants of prehistoric activity, and there is a certain possibility that this activity continued outside of the vicinity of a single site. We can therefore use the KDE not only to localize clusters, but to visualize the intensity in which areas between sites were traversed. To calculate the KDE in a way that reflects land use intensity by travelling between sites, we follow Schmidt et al. [3] in using the mean Nearest Neighbour Distances from the previous NNA analysis as the bandwidth for the quartic kernel, and weighting the sites according to their archaeological composition, i.e. multi- (weight of 2) or single-layered (weight of 1). A comparison of this use intensity model in all three regions and two different time slices allows us to identify differences in land use patterns of Western Mediterranean hunter-gatherer groups.

### Social networks and long-term sustainability

A final step in the analysis arises from the site density analyses. As the archaeological sites represent the remnants of human subsistence activity, we test to see if the distribution of these sites would have the potential to form stable social networks which guarantee the long-term survival of the Pleistocene populations. Thus, a modified version of Wobst’s Mating Network Model [72] was applied to our dataset.

Wobst divides hunter-gatherer populations into minimum bands forming a network with other minimum bands. The purpose of these networks is communication, trade, and ensuring mating partners. Each minimum band comprises of about 25 individuals (cited the “magical number” for group size of hunter-gatherers [73] and is ideally arranged in a hexagonal honeycomb structure with 6 neighbouring minimum bands. Following his simulations, a mating network must comprise of at least 175 individuals, given no mating restrictions, and up to at least 475, with various restrictions. He assumes that societies will hover slightly above this minimum size.

To apply the model to our dataset, we overlaid our sites with different sized hexagonal patterns to simulate inhabitants of archaeological sites forming minimum bands with different range sizes. The functionality of a network is based on having access to enough people in a certain range, meaning that an increase in population density (and a subsequent decrease in range size) should increase the stability of a mating network at least to the point when population growth leads to resource shortage. To assess the social stability of our four archaeological techno-complexes, we simulated networks with steadily increasing artificial population densities (0.01, 0.025, and 0.05 people per km^2^, derived from [74] and corresponding decreasing range sizes to define the threshold at which these networks would be sustainable. If the networks are not sustainable at low population densities, they would be more susceptible to breakdown through external influences.

Stability of the mating networks was analysed by determining the maximum viable travel distance to neighbouring bands and testing if each minimum band had enough neighbours to form a sustainable network. This maximum distance was conceived as the travel distance the bands would have to make under the worst possible conditions of hunter-gatherer adaptations presented by Stein Mandryk [75], the underlying assumption being that travel over these distances would be too costly and lead to a minimum band breaking off to form another network or dying out if no other groups were available [72]. A maximum travel distance of 214.8 km was calculated after the same fashion as Stein Mandryk’s maximum distance of 300 km (citing [76], but taking restrictions on hunter-gatherer lifestyles as variables into account [75]: minimum bands of 15 people with ranges of 2500 km^2^ would need 11 neighbouring bands to sustain a mating network with no mating restrictions; the distance between the two most marginal bands in an optimally packed hexagonal network is 214.8 km. A minimum band of 25 members is considered part of a sustainable network if it has 6 neighbouring bands in a radius of 214.8km, implying open networks and no mating restrictions.

We overlaid our sites with three different hexagonal patterns, the size of the hexagons corresponding to ranges of 25 people inhabiting an area at 0.01, 0.025 and 0.05 people per km^2^. A buffer with a radius of 214.8km was drawn around the centre point of each of these ranges, and the number of neighbours was tallied. If this number exceeded 6, the range was deemed sustainable.

This analysis aims to identify settlement areas more or less susceptible to breakdown of human occupation due to population fluctuations. As this, much like the previous spatial analyses, relies mainly on the presence or absence of sites, a high-quality database is critical. Although we do not assume to have a dataset representing the complete prehistoric activity on the Iberian Peninsula and in Morocco, we regard the data as adequately robust for this analysis. By modelling social networks in this fashion, we aim to examine the long-term habitat stability of the different macro-regions of the Western Mediterranean, adding a crucial facet to our multi-proxy analysis of prehistoric land use patterns.

## Results

### ^14^C-Chronology

**Fig 4:**
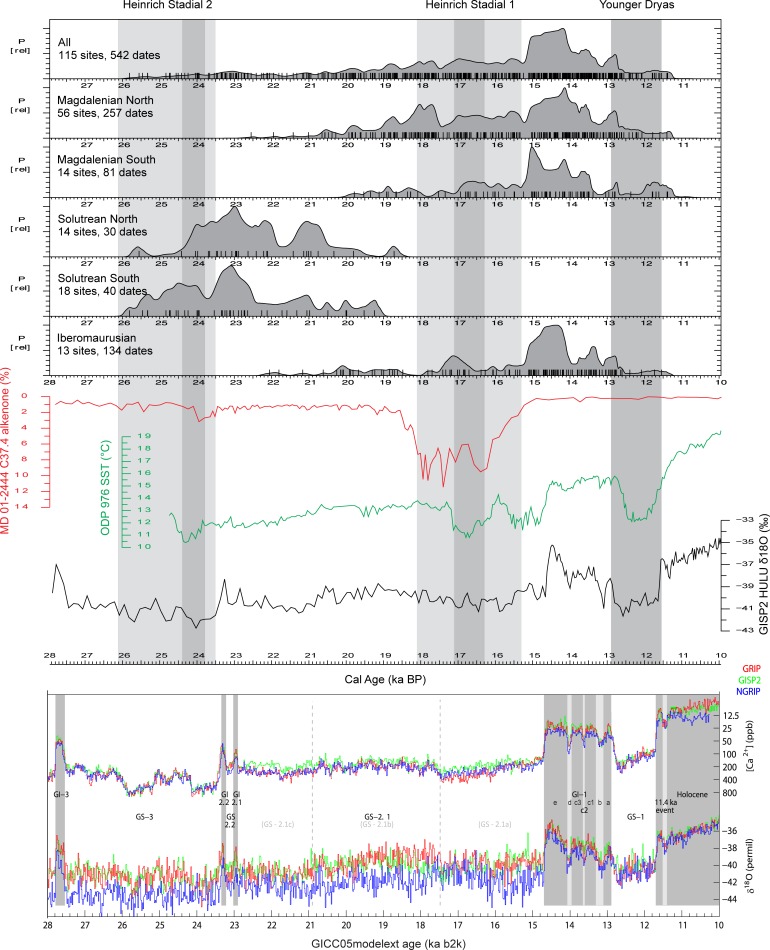
Summed probability curves of all calibrated radiocarbon dates from Solutrean, Magdalenian and Iberomaurusian sites in the Western Mediterranean, together with climatic data for the Late Glacial period. The upper climate curve (Cold Water surges) shows the southwards shift of the North Atlantic ice as recorded by C37.4 alkenone of core MD-01 2444 [77]. The climate curve in the middle reflects the variation in sea surface temperature of the Mediterranean Sea as reconstructed from relative proportions of di- and tri- alkenones of core ODP-976 [78]. The lower curve shows the δ^18^O record of the GISP2 ice core [79]. Grey columns display the timing of Heinrich Stadials 2 and 1 and their subdivision in an early, main and late phase as suggested by the pollen record of Alborán Sea core MD95-2043 [80]. The temporal limits for the Younger Dryas (11.7 to 12.9 ka BP) are taken from the recent compilation of delta^18^O and Ca^2+^ records of Greenland ice cores [1]. The SCDPD curve of the Western Mediterranean matches climate change from the Greenland intimate curve [1]. Unfavourable climate conditions during cold phases are reflected by drops of the SCDPD curve.

**Fig 5:**
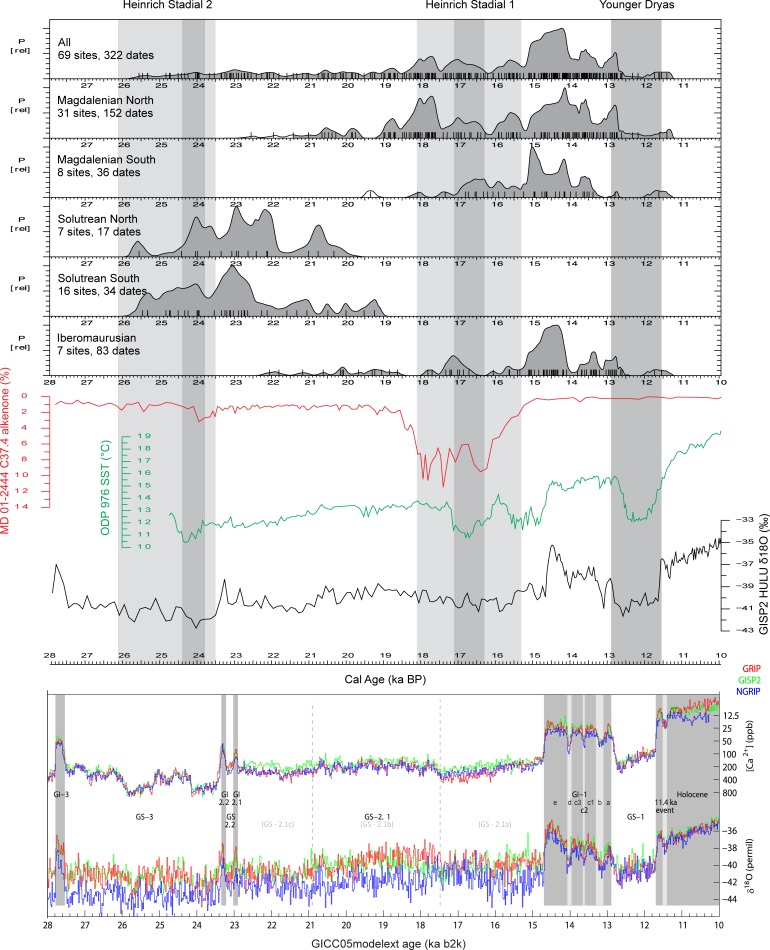
Summed probability curves of calibrated AMS radiocarbon dates only from Solutrean, Magdalenian and Iberomaurusian sites in the Western Mediterranean, together with climatic data for the Late Glacial period.

Our dataset comprises a total of 542 dates from the Western Mediterranean separated by techno-complex and region (Fig 4, S2 Table). This is refined in Fig 5, which displays only the 322 AMS-dates. Although we processed more than 500 14C dates in the summed probability curves, we have to take into account that the representativity might be below a confident threshold, as our dates span more than 10ka and come from a very large area.

As we are studying land use differences on the supra-regional scale, short term fluctuations identified in the 14C plots–resulting from dating biases or reflecting actual trends in occupation history–are difficult to detect in the following analyses due to the necessary coarse resolution, but will be discussed in the concluding chapters of this paper.

The Solutrean commences at the onset of the cold and dry HS 2 after 26 ka calBP. During this initial phase, a particularly high number of dates are recorded in the southern part of Iberia, while evidence in the North is quite weak and only increases towards ~24 ka calBP. For the whole Iberian Peninsula, a data peak is recorded at around 23 ka calBP. After 20 ka calBP, the Solutrean comes to an end within GS-2.1b. In general, the Solutrean data distribution displays a very similar pattern in both regions. This changes during the Magdalenian. The transition to the Magdalenian appears to be a progressive phenomenon, with a first patchy occurrence of Magdalenian layers before 20 ka calBP solely in the northern territories. Here, evidences of both techno-complexes chronologically overlap. In contrast, the appearance of the Magdalenian in Southern Iberia is slightly delayed, and first records are only dated to the time frame between 20 and 18 ka calBP. Moreover, when only AMS dates are concerned (Fig 5), a gap in human occupation during the transition from the Solutrean to the Magdalenian seems probable. The time span between 20 and 15.5 ka calBP experiences the development of stable Magdalenian occupation in Northern Iberia, in contrast to Southern Iberia. Before 15.5 ka calBP, the Magdalenian occupation is much weaker here. A peak phase starts only after 15.5 ka calBP, corresponding to far-reaching increases of temperature and humidity during the Greenland Interstadial 1. The end of the Magdalenian occupation is marked in both areas of Iberia by the cold Greenland Stadial 1 (Younger Dryas).

The Iberomaurusian in Morocco appears after HS 2, with first evidences around 22 ka calBP. However, compared to Southern Iberia, no peak phase is apparent such as in the Solutrean. For the first seven millennia, the signal for human occupation is very low in the Early Iberomaurusian (during GS-2.1a-b-c). Archaeological evidences are indeed limited to few sites, which are mainly concentrated in the mountainous North-East. Only during HS 1 does Morocco display a first slight increase of Early Iberomaurusian occupation. After HS 1, during the Late Iberomaurusian, the strong increase of data corresponds to a general increase in site numbers and to a development, in many locations, of rich and thick shell midden deposits, so called “escargotières” [25]. The number of 14C dates drops around 13–12 ka calBP, during the Younger Dryas, marking the end of the Iberomaurusian. Overall, both regions of Iberia experience very similar peak phases during 26–20 ka calBP while the data record in Morocco points to an extremely low-scale human occupation. During HS 1 and the Younger Dryas, a disruption is visible in the entire Western Mediterranean. The radiocarbon record supports a correlation between human occupation density and climate change in the Western Mediterranean, although different regional climate regimes can be expected. This requires further analysis of other local datasets.

The Early Iberomaurusian overlaps with the Solutrean and the first part of the Magdalenian. During this phase, the Moroccan radiocarbon record is at its weakest, while the contemporaneous Solutrean in Iberia shows intensive human occupation. This changes after the LGM, when Southern Iberia now evidences weak human occupation until the end of HS 1, while the data increases slightly in Morocco. The record in northern Iberia is only very slightly affected by the breakdown of radiocarbon data seen in the South. After HS 1, all regions experience a peak phase of radiocarbon data, which breaks down again during the Younger Dryas.

The chronology of the techno-complexes under study is not synchronous from Iberia to Morocco. This makes the comparison between the four techno-complexes difficult, as they overlap slightly. However, a crosscheck between Southern Iberia and Morocco is of special interest because we expect similar dry environmental conditions on both sides of the Strait of Gibraltar. For this reason, the following results from both sides of the Western Mediterranean will be discussed together, but all direct comparisons must keep this temporal overlap in mind.

### Site numbers, archaeological composition and distribution

**Fig 6:**
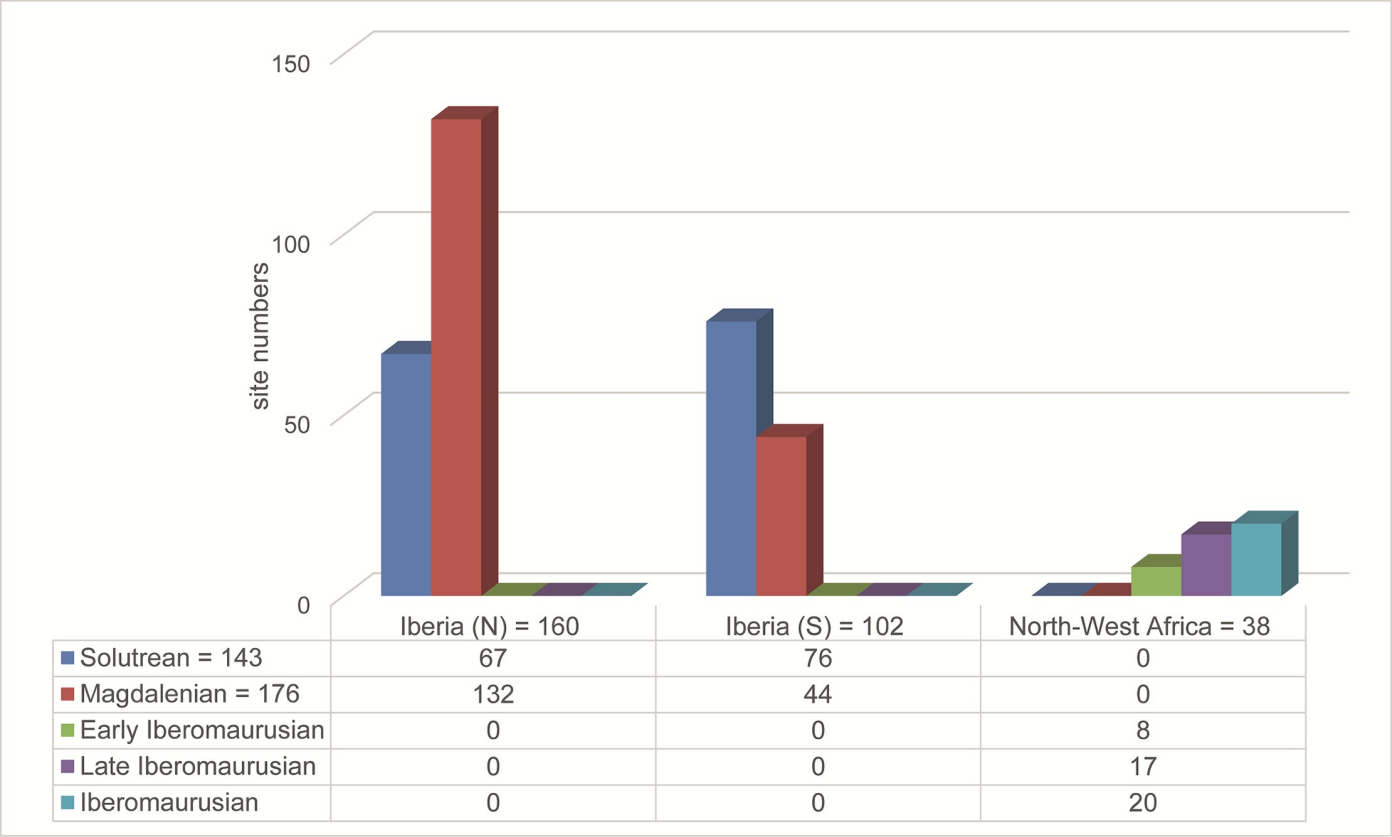
Site numbers for the regions Iberia (north), Iberia (south) and Morocco per techno-complex Solutrean, Magdalenian, Early and Late Iberomaurusian. Category “Iberomaurusian”: the attribution to the Iberomaurusian is clear, but the period (Early or Late) is unknown due to lack of dating. As many sites were occupied during multiple techno-complexes, the sum of the site numbers in the data table do not add up to the total site numbers.

The regional and diachronic distribution of the 300 archaeological sites in our database shows two prominent trends: an increase of site numbers from early to late periods in Northern Iberia and Morocco, and a decrease of sites in Southern Iberia (Fig 6). While the number of Solutrean sites is higher in the South than in the North, the opposite is visible in the Magdalenian. In the North, the site numbers increase drastically in the later phase, while they decrease in the South. In North-West Africa, a general trend of an increase of site numbers from early to late sites is visible as well. This strong fluctuation of site numbers indicates changes in the settlement system in Iberia and to a lesser extent in the Iberomaurusian of Morocco.

**Fig 7:**
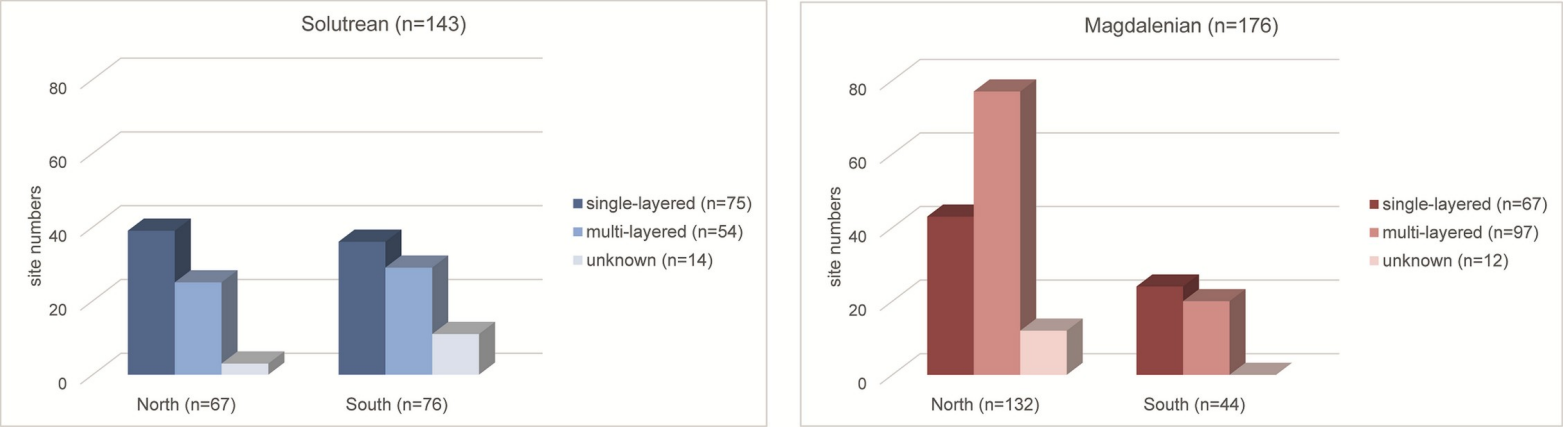
Comparison of the regional distribution of single- and multi-layered sites during Solutrean and Magdalenian on the IP. Category “unknown”: the attribution to Solutrean/Magdalenian is clear, but there is no information on the number of stratigraphic units.

To get further insight into settlement dynamics, we compare the distribution of single- and multi-layered sites in the different regions of the IP (Fig 7). As described above, no separation between single- or multi-layered sites could be undertaken for the Iberomaurusian, and the comparison only takes the Iberian sites into account.

During the Solutrean, settlement dynamics in Iberia seem quite balanced in both the North and the South; single-layered sites are the most frequent category in both regions, and the relationship between single-layered and multi-layered sites is similar. This indicates comparable conditions on the whole IP. During the Magdalenian, the situation is noticeably different. The boom of Magdalenian sites in the North is accompanied by a boom of multi-layered sites. In Southern Iberia, the general decrease of site numbers does not change the relationship between single- and multi-layered sites. Both changes in site numbers and archaeological composition point to the emergence of a new settlement system in Iberia during the Magdalenian.

In a second step, the re-use of sites and settlement areas in succeeding techno-complexes are analysed (see Fig 3). 39 of the 160 sites in the northern Iberian Peninsula were occupied during both techno-complexes (24.38%), while in the southern Iberian Peninsula this is only the case for 18 of the 102 sites (17.65%). In Morocco, 7 of the 38 sites have multiple techno-complexes (18.42%); of these, 5 are also multi-layered. Major changes in land use systems seem to have occurred in Southern Iberia and Morocco, while the high rate of site re-occupation in Northern Iberia provides another argument for long-term settlement stability in this region.

In addition to this, few sites with occupations during multiple techno-complexes are multi-layered in both time frames (10 in the northern Iberian Peninsula, 5 in the southern Iberian Peninsula and 5 in the Iberomaurusian). In the North, only one of these sites (Cardina I) is located in the interior of the Peninsula, while the other 9 are along the Cantabrian Coast, a region densely settled during both time frames. In the South, 4 of these sites are also located in large clusters on the Atlantic and Mediterranean coasts, with only Nerja being more isolated. In Morocco, 3 of the 5 multi-layered sites from multiple techno-complexes are located in the Eastern Rif, another densely inhabited region, while the other two are located close to neighbouring sites (Kehf el Hammar and Dar es-Soltan 2). Such sites, often amidst clusters of other sites, therefore seem to play a special role in the settlement system.

In conclusion, multiple changes in settlement pattern during the Western Mediterranean Late Pleistocene are apparent. Before and during the LGM, site numbers are high in Northern and Southern Iberia, while they are extremely low in Morocco. After the LGM, in the Magdalenian, site numbers increase significantly in Northern Iberia as well as in Morocco, the latter albeit on a much smaller scale, while they decrease markedly in Southern Iberia. This increase of site numbers in Northern Iberia goes hand-in-hand with an increase of multi-layered sites, while the relationship between single-layered and multi-layered sites in the South remains unchanged. In addition to this, more Solutrean sites in the North are reoccupied during the Magdalenian. Sites that yield multi-layered occupations during both techno-complexes are regularly located in the middle of site clusters and along the coasts, and probably represent core areas of human occupation.

### Site distribution and land use

For the following density analyses, the Early and Late Iberomaurusian time frames were combined. As outlined above, only a few sites in our database could be attributed to the Early Iberomaurusian, so that statistical analyses are not expected to yield reliable results. Most of these sites were reoccupied in the Late Iberomaurusian, and there is a large number of sites where the attribution to either phase is unclear. For this reason, all Iberomaurusian sites were analysed together for the Nearest Neighbour and Ripley’s K analyses.

The results of the Nearest Neighbour Analyses are presented in Table 1. Due to large differences in the size of the study region, the nearest neighbour statistics cannot be compared over the three regions (marked by the thick lines in Table 1); we can only directly compare the diachronic changes in one region. However, the observed mean distances, i.e. the mean of the measured distances between the nearest neighbours, can be compared. These results display the amount of clustering that can be observed in the dataset; the smaller the Nearest Neighbour Ratio, the stronger the clustering.

**Table 1:** Nearest Neighbour Statistics of the Late Pleistocene Settlement in the Western Mediterranean.

Timeframe and Region	n	Observed Mean Distance (km)	Expected Mean Distance (km)	Nearest Neighbour Ratio	z-Score	p-value	Interpretation
Solutrean (N)	67	11.859	34.602	0.342734	-10.2922	0	significantly clustered
Magdalenian (N)	132	13.297	24.652	0.539385	-10.1241	0	significantly clustered
Solutrean (S)	76	14.112	29.362	0.480598	-8.6625	0	significantly clustered
Magdalenian (S)	44	18.840	38.590	0.488203	-6.4946	0	significantly clustered
Iberomaurusian	38	31.604	56.060	0.565014	-5.1298	0	significantly clustered

Number of analysed sites n by timeframe with Observed and Expected Mean Distances in km, Nearest Neighbour Ratios, z-scores and p-values as provided by ArcGIS 10.3.

All site groups are significantly clustered. In the northern Iberian Peninsula, the Solutrean is markedly more clustered than the Magdalenian, evidencing a clear difference in settlement pattern. The increase of Magdalenian sites goes hand-in-hand an expansion of settlement areas. In the southern Iberian Peninsula, the values are almost identical, demonstrating that the decrease of Magdalenian sites did not change the overall settlement structure. In Iberia, the observed mean distances are in higher in the South and especially in the Magdalenian, pointing to an increased mobility compared to Northern Iberia. The Iberomaurusian, with the fewest number of sites, also shows significant clustering, but displays the largest observed mean distance of all techno-complexes. This finding suggests highest mobility in the Iberomaurusian.

**Fig 8:**
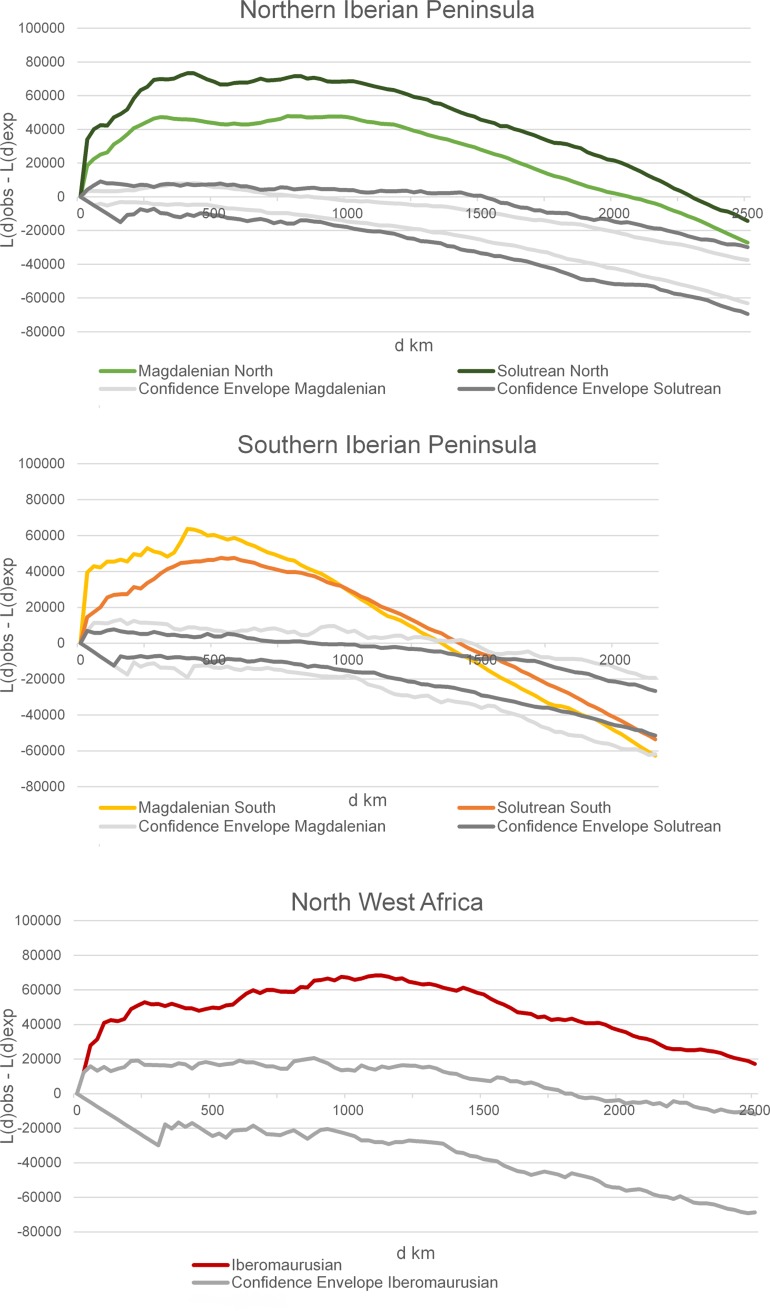
Ripley’s K analysis of sites by region.

To follow this, we did Multi-Scale Ripley’s K Cluster Analysis (Fig 8). This gives us an indication of the cluster size as well as the nature of the patterning. In general, we note two types of curves: a curve with two peaks in the northern Solutrean and Magdalenian as well as the Iberomaurusian, and a steep curve with its highest point at small distances for both techno-complexes in the southern Iberian Peninsula. The former represents small clusters forming large clusters, in contrast to the steep curves in the South, which reflect cluster isolation. The more intense clustering in the North during the Solutrean than during the Magdalenian is confirmed, as the low values of the Magdalenian curve indicate a relatively homogeneous distribution. In Southern Iberia, the sites also make up small and then somewhat larger clusters, but the curves peak at very small distance band values and the clusters become increasingly isolated from each other at larger distances. Here, the Magdalenian demonstrates stronger clustering than the Solutrean, as opposed to the North. While the settlement pattern in the North shifts towards homogeneity over larger areas, the clustering in the South increases. These clusters are now farther apart, and settlement becomes inhomogeneous. Differences in the settlement pattern between the North and the South are therefore very clear. The Iberomaurusian expresses a similar curve pattern as the northern Iberian Peninsula, showing clustering on small as well as on large scales, different from the pattern in southern Iberia; we should however keep in mind the large differences in site counts between northern Iberia and Morocco.

To translate these results onto a map and analyse the geographical distribution of the site clusters, we calculated the KDE. The KDE bandwidth was derived from the mean of all mean nearest neighbour distances (17.942 km); the resulting figures can be interpreted as models of the intensity of human influence on the landscape based on inter-site mobility.

Following the previous discussion of multi- and single-layered sites, we think it adequate to incorporate a weighting factor into the model to reflect more or less intense use in accordance to the archaeological data. Multi-layered sites are weighted with 2 instead of the default weighting 1, to reflect repeated occupation of the site by people during one techno-complex. For the Late Iberomaurusian, the situation is somewhat more complicated. Due to the more rapid sediment accumulation of the escargotière, a multi-layered Iberomaurusian site is not comparable to a multi-layered site from the Iberian Peninsula. Because of this, we derived more intense use from the thickness of the escargotière in published site stratigraphies. Settlement layers thicker than our (arbitrary) limit of 50cm were weighted like multi-layered sites with a value of 2; all other sites had the same default weighting as single-layered sites. Sites without reliable information on stratigraphy were weighted as a single-layered site. The unsure dating of the Iberomaurusian is also problematic. As we aim to examine the increase in site numbers from the Early to Late Iberomaurusian, we cannot combine the time frames as we did in the previous site density analyses. To incorporate this problem in our model, instead of grouping all Iberomaurusian sites into one, we recognize the uncertainty and display all “unknown” sites with half transparency next to the securely dated sites without transparency. The calculations were however undertaken together.

**Fig 9:**
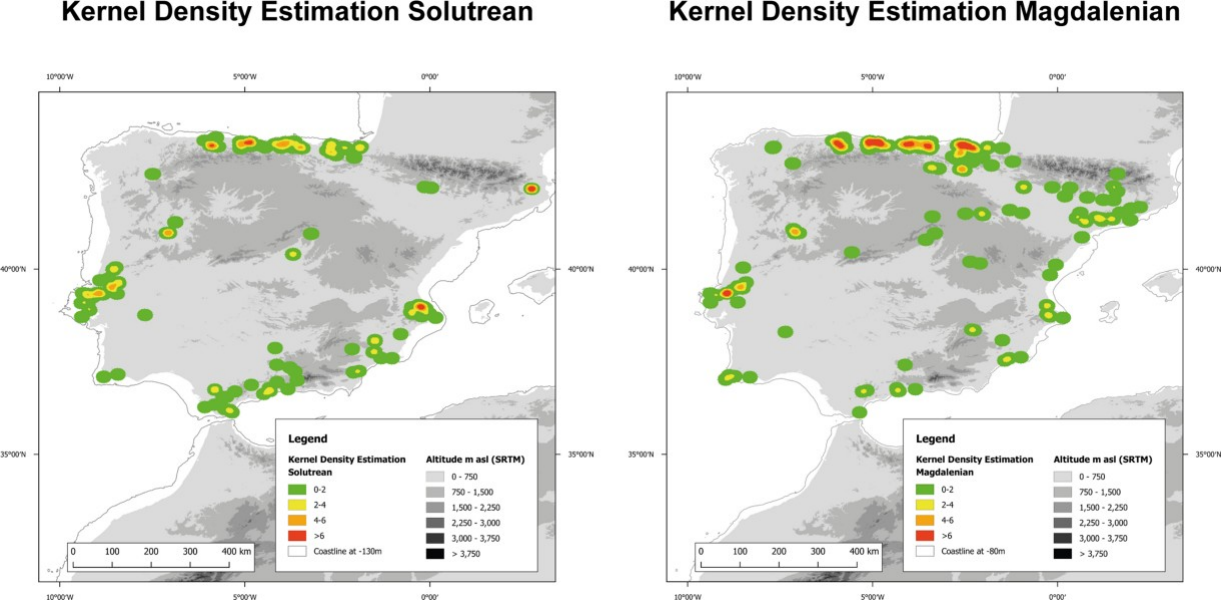
Kernel density estimate of the Solutrean and of the Magdalenian in Iberia. Included are Palaeocoastlines of -120m for the Solutrean and of -80m for the Magdalenian [81]. Digital Elevation Model from [82].

**Fig 10:**
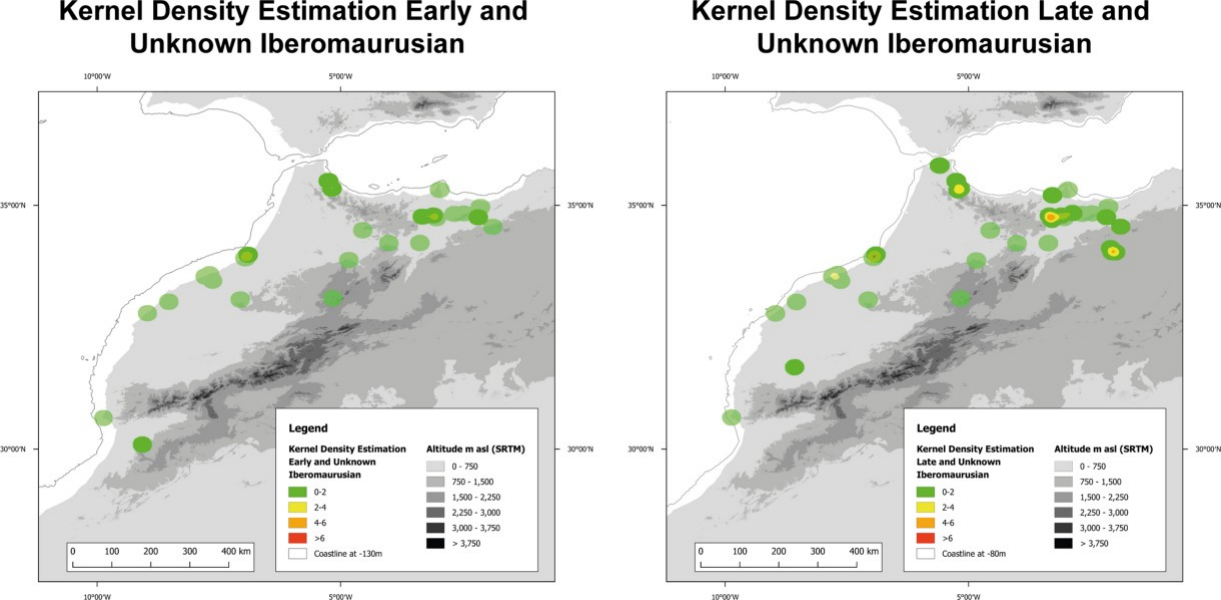
Kernel density estimate of the Early/Unknown Iberomaurusian and of the Late/Unknown Iberomaurusian in Morocco. Included are Palaeocoastlines of -120m for the Early/Unknown Iberomaurusian and of -80m for the Late/Unknown Iberomaurusian [81]. Digital Elevation Model from [82].

Using Qgis 2.8, we calculated a bivariate KDE for each time frame separately and combined them into one diagram. The result is shown in Figs 9 and 10.

In the Solutrean (Fig 9), we can see multiple areas of dense site clusters along the Northern coast, along the Southern Mediterranean coast and in Portugal. Only isolated clusters and sites show up in Central Iberia. In the Magdalenian the pattern changes. In the North, settlement disperses into the Interior and the North-East while a simultaneous increase in activity on the northern coast is documented. The site distribution shows a rather homogeneous use of a larger territory. The opposite is visible in the South: large stretches of land are now abandoned, and the sites form much smaller, tighter clusters. On the western Atlantic coast, density values remain on the high level of the preceding Solutrean, while they decrease and condense on the eastern Mediterranean coast. This reflects continuity of human activity in the West and decreased human activity in the South-East.

In Morocco in the Early Iberomaurusian clustering is low (Fig 10) and we can see a very scattered distribution of sites in coastal areas as well as in inland areas, even when we consider the relatively high number of multi-layered sites in this group. This means that during the LGM, Early Iberomaurusian occupation intensity was probably very low. The Late Iberomaurusian seems to experience a relative increase of human activity when compared to the earlier phase. Although site distribution is still very dispersed, more regions with more intense settlement activity can be identified. The Eastern Rif and the coastal regions are occupied more homogeneously, while the settlement remains scattered in other areas. The general increase of sites from Early to Late Iberomaurusian and the appearance of some strong clusters is particularly notable.

The site density analyses show clear differences in land use pattern over time and space in Iberia. In the North, settlement expands and becomes more homogeneous through time, while it is more constricted in the South. In North-West Africa, settlement structure is very dispersed with low site density and gets more stable only in the Late Iberomaurusian; this difference to Southern Iberia will be subject to further discussion.

### Social networks and settlement sustainability

**Fig 11:**
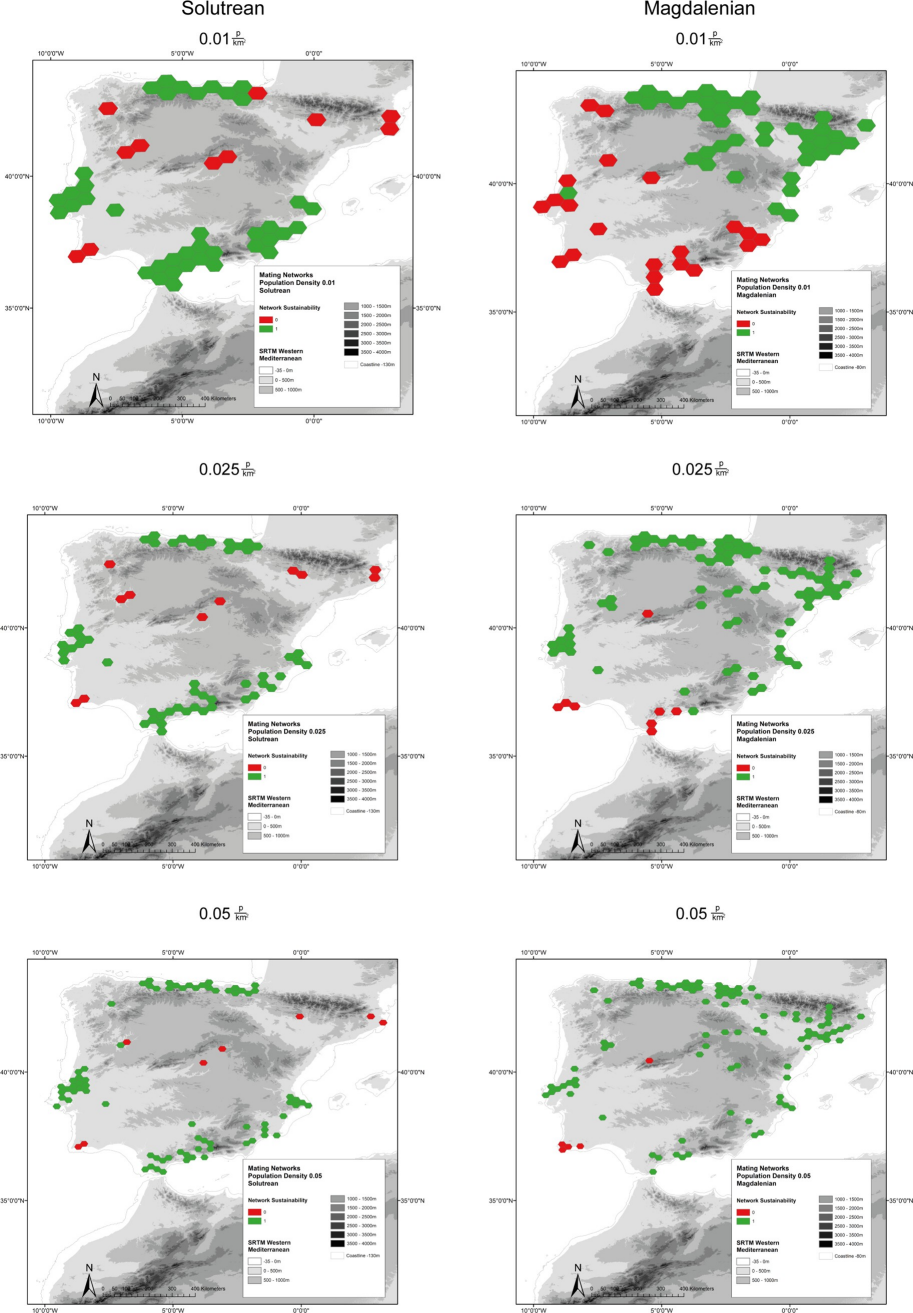
Mating Networks of Solutrean and Magdalenian settlement in Iberia with population densities of 0.01, 0.025, and 0.05 people per km^2^. Included are Palaeocoastlines of -120m for the Solutrean and of -80m for the Magdalenian [81]. Digital Elevation Model from [82].

**Fig 12:**
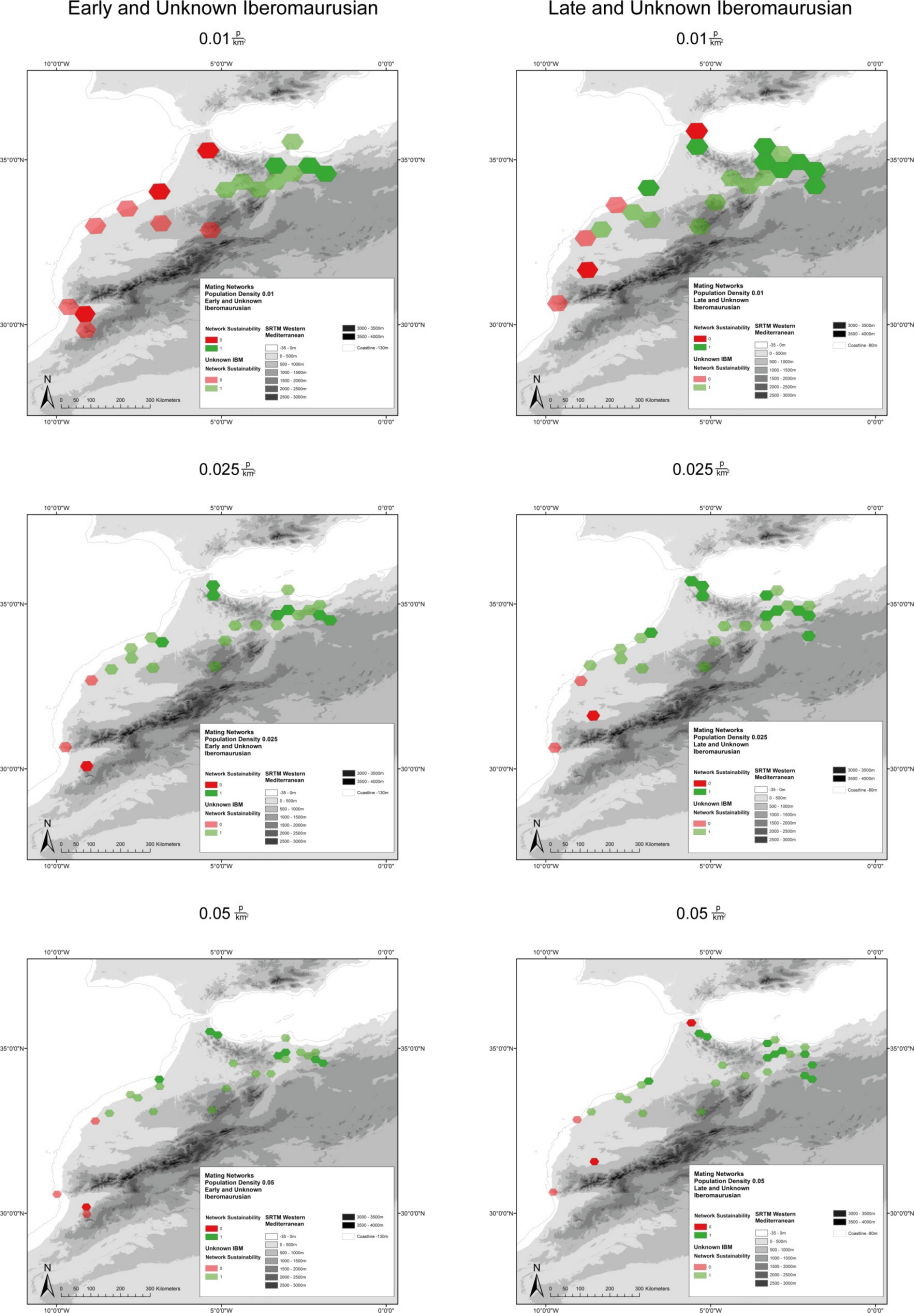
Mating Networks of Early/Unknown and Late/Unknown Iberomaurusian settlement in Morocco with population densities of 0.01, 0.025, and 0.05 people per km^2^. Included are Palaeocoastlines of -120m for the Early/Unknown Iberomaurusian and of -80m for the Late/Unknown Iberomaurusian [81]. Digital Elevation Model from [82].

To study possible effects of these changes in settlement pattern on the population, we applied the Mating Network Model (Figs 11 and 12). Multiple networks were simulated with different range sizes and population densities in order to identify a threshold at which networks could have been sustainable. Again, unknown Iberomaurusian sites are displayed with half transparency.

The model under the worst conditions with the highest range and smallest population density of 0.01 people per km^2^ shows large stable networks during the Solutrean in all settlement areas except for the Interior (Fig 11). This changes dramatically in the Magdalenian. Southern Iberia including Portugal disintegrates into patchy isolated settlement areas and small, unstable networks. However, in contrast to the preceding Solutrean, the Magdalenian networks in the North now expand into Central Iberia. A clear division roughly around 40° Northern Latitude from the North-West to the South-East through the Iberian Peninsula can be seen. Only one band below this line could theoretically form a stable network; however, as this network is comprised of unsustainable minimum bands, its lifespan would be limited to that of the surrounding bands. It is clear that under the worst conditions, the Magdalenian in the South would not have been sustainable in the long-term. With a simulated increase in population density to 0.025 people per km^2^, the pattern on the Iberian Peninsula during the Solutrean remains unchanged. The Magdalenian fares much better, although bands in the far south would still not be stable in the long term. Only at a population density of 0.05 people per km^2^ does the Magdalenian settlement becomes stable over most regions of the Iberian Peninsula, with only the far South-West and a part of the Interior as an exception. The relatively high threshold of 0.025 to 0.05 people per km^2^ indicates that Southern Iberia was probably unstable for human settlement during the Magdalenian.

In Morocco, at a population density of 0.01 people per km^2^, the Early Iberomaurusian forms stable networks only in the eastern regions (Fig 12). The situation gets better during the Later Iberomaurusian, but the western area along the Atlantic coast remains highly vulnerable, especially in the southernmost parts. Interestingly, the northernmost band towards the gateway to the Iberian Peninsula may also be rather fragile; further examination is needed to address this observation. This might be an important clue for future investigations on the contacts via the Strait of Gibraltar.

## Discussion

### Changes of settlement pattern and land use

Different methods of our multi-proxy analysis support the results of previous studies on population and land use dynamics the Western Mediterranean [3,4,7,11,14,25,33]. The decrease in human presence in Southern Iberia was already noted by Straus et al. [14], and the increase in sites from the Early to Late Iberomaurusian was recognized in Linstädter et al. [25]. Numerous studies have demonstrated the fragility of Upper Palaeolithic groups in Europe due to very low population densities [7,83,84], resulting in highly vulnerable settlement structures and land use patterns.

In our study, we can observe multiple trends concerning settlement pattern and human land use in the Western Mediterranean. The number of sites and their distribution in space changes in a diachronic as well as a regional perspective. On the Iberian Peninsula, differences in the settlement pattern between the North and South become visible. The Solutrean in both areas of Iberia is represented by a high number of sites. The stratigraphic composition of these sites is characterized by a slight predominance of single-layered occupations in both areas; this is somewhat stronger in the North [3]. This regional balance gives the impression of similar settlement systems in Northern and Southern Iberia, especially in the coastal areas, while the occupation of the Interior is weak [3,26]. Although the number of sites is slightly higher in the South, the mean distance of sites from the Nearest Neighbour analysis is somewhat larger. This might point to higher mobility of hunter-gatherer groups and could reflect slight differences in settlement systems in Iberia [85,86]. Faunal analyses support differences in the Solutrean of both areas as well [87]. The steep curve of the Ripley’ K analysis in the South reinforces this impression and shows patchier settlement in the South than the North. However, in the Kernel Density Estimate, large regions which were inhabited homogeneously can be identified, and the Mating Network Model results in stable social networks and low system vulnerability, despite this patchiness. Solutrean settlement was probably stable in Northern and Southern Iberia, with the exception of the Interior and the North-East.

The situation changes significantly with the Magdalenian. In the Northern Iberian Peninsula, radiocarbon chronology of the last Solutrean sites and the early Magdalenian sites overlaps slightly, while in the South, a gap between both techno-complexes seems probable. This chronological discontinuity might indicate population discontinuity as well, and the South may have been less attractive for human settlement. The most striking feature is a dramatic decrease of Magdalenian sites in the South, while in the North, site numbers almost double and many locales are reoccupied. This indicates increased human activity in the landscape, marking, without a doubt, a clear change in the settlement system. For the first time, a predominance of multi-layered sites is documented in the Magdalenian in Northern Iberia. Interestingly, sites in the South are particularly clustered, while they are more uniformly distributed in the North. Another important element of settlement system and demographic development is displayed by the result of the mating network models: in the North, networks were probably very stable during the Magdalenian, while they were very unstable in the South. The strong coastal orientation of settlements stretches the societies’ territories and complicates the development of stable mating networks. The distribution of radiocarbon dates supports weak human occupation after the LGM und during HS 1 in Southern Iberia. Only after HS 1 does the data curve increase here again.

In Morocco, the Early Iberomaurusian occupation starts significantly after HS 2, during the final phase of the Solutrean in Iberia, and runs parallel to the beginning of the Magdalenian. Site numbers are low and site distribution is therefore difficult to interpret, but foremost seems to indicate scarce human presence. This is evidenced not only by the site numbers, but also by the results of the Mating Network Model. Interestingly, many of the Early Iberomaurusian sites are multi-layered. This late appearance and the low human presence of the Iberomaurusian compared to the Solutrean of Southern Iberia merits an explanation. We assume that after a major gap in human occupation after the Middle Palaeolithic [25,32], repopulation of Morocco started only slowly, and development of a stable human population took some time.

Radiocarbon chronology shows a slight increase of dates during HS 1, with the end of the Early Iberomaurusian, which then increases significantly after HS 1 in the Late Iberomaurusian. Most Early Iberomaurusian sites are reoccupied during this phase; the site pattern is similar to that of the northern Iberian Peninsula during the Magdalenian (Fig 8). The Kernel Density Estimate also shows a clear increase in human activity and a homogeneous settlement pattern, which forms stable social networks in most areas of Morocco. These results indicate a continuous settlement pattern from the Early to the Late Iberomaurusian. Compared to the Early Iberomaurusian, these changes could be interpreted as an increase in human presence.

Despite Morocco and Southern Iberia being very similar to each other in both climate and vegetation, we can see clear differences between the two regions. We assume that these are an expression of differences in human behaviour. Resource availability, dispersion and density are crucial for the survival of hunter-gatherers and have an impact on mobility and land use [73]. Besides economic mobility, social mobility plays an important role in hunter-gatherer societies and demography as well [88]. At the moment, it is unclear which features triggered the opposite trends evident in the Western Mediterranean. The changes in the settlement system in the Western Mediterranean took place at the end of the LGM and immediately after HS 1; can these discrepancies be traced back to environmental change, or is there an indication for internal cultural adaptations?

### Climate change as cause of settlement disparities?

The radiocarbon curves for Southern Iberia and Northern Morocco closely trace climate oscillations from HS 2 until the Younger Dryas. Therefore, the question arises whether climate conditions were the main factor behind diachronic changes in Palaeolithic land use and settlement pattern in the Western Mediterranean. Several terrestrial and marine geological archives yielded detailed and chronologically well-constrained palaeoclimate proxy records [18,19,89], providing evidence for climate change in Iberia and North-West Africa during the period of interest.

Most proxy records suggest that the LGM was colder and drier than today. The reduction in moisture is, for instance, clearly documented by increased percentages of semi-desert taxa (*Artemisia*, *Chenopodiaceae*, *Ephedra distachya*) and steppe vs. forest vegetation in pollen records of Padul [90] and the Alborán Sea (MD95-2043 [80]; ODP976 [91]). Dry climatic conditions may also be deduced from the formation of clay dunes in the Manchega Plain, dated to the time frame between 18 and 28 ka [36]. Pollen records of core MD95-2042 retrieved from the Iberian shelf off southern Portugal suggest that the LGM was less cold and dry than the end of MIS3 or than stadial periods correlating with Heinrich Stadials [92]. However, high percentages of *Ericaceae* and *Pinus* point to relatively humid conditions not necessarily dryer than today. The d^18^O-speleothem record of Eagle cave, located in Central Iberia, suggests that the LGM was drier than the end of MIS3, when Iberian glaciers reached their maximum extent [93]. The same record shows peaks in less negative values, indicating aridity during HS 2 and HS 3. Global climate models included in the Palaeoclimate Modelling Intercomparison Project (PMIP3) simulate higher precipitation for the LGM than for the pre-industrial period in the Western Mediterranean, as explained by southward migration of the North Atlantic Jetstream and related storm tracks [21,22]. However, very limited evidence for this positive anomaly is found in palaeoclimate archives, and the notion of dryer and colder conditions than today appear to be more likely, according to the present state of knowledge.

HS 1 [16] was probably much drier and colder than the LGM, as indicated by maxima of the cold-water foraminifer *Neoglobigerina pachyderma (s)* and in ice rafted debris (IRD), concomitant minima in reconstructed sea surface temperatures (SST), e.g. [78], and increases in cold- or arid-tolerant pollen. Interestingly, the pollen spectra for HS 1 both in marine cores off North-West Iberia [23] and in the Alborán Sea [80] show a tripartite pattern with a peak in continental dryness during the second phase between about 17.1 and 16.3 ka. This roughly 800 year-long phase of maximum dryness is not well recorded in terrestrial archives, however, possibly due to problems in resolution and chronological dating. The Greenland Interstadial 1, a phase of climate amelioration, is well represented in pollen spectra, reconstructed SST and other climate proxies. At Padul, it is characterized by a clear spread of *Quercus* type pollen and a reduction in *Artemisia*, a regional climatic pattern, which is closely mirrored in the MD95-2043 core. After GI-1, the climate got dryer and colder during the Younger Dryas, clearly reflected by multiple records covering the Late Glacial/Holocene transition in northern and southern Spain. The general picture of cold and arid climatic conditions during HS 2, HS 1, and the Younger Dryas (YD), interrupted by comparatively moist phases of the LGM and GI-1, is also reflected in proxy data for Northern Iberia [19,23,94,95].

The climatic conditions in North-West Africa are less well known, but proxy records from marine cores retrieved from the North-Western African margin suggest that HS 1 was the driest period between HS 2 and the Younger Dryas in North-West Africa [96–101]. Less information is available from terrestrial climate archives in North-West Africa. According to proxy data from Lake Ifrah, located in the Middle Atlas, a comparatively wet phase from 29 to 24 ka was followed by a dry phase [24 to 12 ka) and again a wet phase [12 to 5 ka) [102]. However, clear signals for Heinrich Stadials were not detected. This is also the case for pollen records of Ait Ichou [103] and Ras-El Ma [104]. At Lake Ifrah [102], Lake Tigalmamine [105], and Ait Ichou [103], dry and possibly cold climatic conditions probably prevailed until the early Holocene, as indicated by high *Artemisia* percentages. The Younger Dryas is apparently not represented in the pollen diagrams of Northern Africa. The speleothem of La Mine, Tunisia, shows an abrupt onset of the YD with a decrease in delta^13^C, suggesting a colder climate than afterwards. Its continuous growth does not point to drier climatic conditions than before or after the YD event [106]. So far, the documented palaeoenvironmental change in North-West Africa seems to have been less strong than in Southern Iberia.

Although a long tradition deals with cost-benefit ratios of hunter-gatherer subsistence, it remains difficult to define thresholds at which environmental constraints become too risky for human occupation and hunter-gatherer resilience collapses [56,107]. Such breakdowns of hunter-gatherer populations are clearly documented during the LGM in Central Europe, when large areas turned out to be high risk environments and were abandoned by humans [11,108,109]. A similar process is evident at the end of Middle Palaeolithic for Central Iberia [26,110,111] as well as other areas of the Iberian Peninsula [3,112,113]. In Morocco, a population breakdown after the Middle Palaeolithic is also evident [25].

Considering the LGM in Iberia, it apparently did not cause a gap in human occupation. On the contrary; although human presence in Central Iberia, the North-East and the North-West was weak during the Solutrean, the rest of the Peninsula experienced an increase in human presence in the landscape and in stable settlement systems. Considering site numbers and distribution, this changes dramatically during the Magdalenian in the South.

Proxy data and palaeoclimatic modelling shows that dry spells during HS 1 considerably increased the extension of dry areas from the South-East to the rest of Southern Iberia, only leaving small refugia of more favourable conditions [24]. During HS 1, the extent of drylands increased particularly during the summer months (June to August), turning the major part of Southern Iberia into devastating ultrahyperarid areas. We hypothesize that this process had a crucial impact on the settlement system in Southern Iberia, but only insignificant impact on Northern Iberia. Two major site clusters during the Solutrean in the South, which both represent core areas of human settlement, are located in the South-East and in Central Portugal. While the south-eastern cluster shrinks dramatically during HS1 going into the Magdalenian, the cluster in Portugal remains mostly intact. Higher humidity values from to the nearby Atlantic coast could be a reason for this. Although summed probability curves of radiocarbon dates show an increase of Magdalenian data in the South after HS1, this is not matched by site numbers, which still point to a low presence of human groups. We hypothesize that at least the eastern cluster and the large dry areas of the southern Meseta were considerably affected by dry climatic conditions during HS 1, preventing early colonization by Magdalenian groups due to shortage in water, prey, and natural vegetation for nutrition for both humans and animals. The most arid area of Southern Iberia is located in the South-East; as the coastal stretching of settlement areas is a major weakness for mating networks, strong dry spells during HS 1 could easily have interrupted these networks along the coast and have caused a breakdown of regional populations. In addition to this, we hypothesize that the significant increase of Magdalenian sites in the north-eastern regions of Iberia could have been caused not only by a spread of the settlement areas from the northern Atlantic coast to the East, but also by a retreat from the south-eastern areas along the Mediterranean coast to the North.

In Morocco, after a gap in human occupation following the end of Middle Palaeolithic, radiocarbon data increases steadily from the LGM into HS 1. From a very low presence of humans in the LGM, stable clusters seem to have developed in the course of the Early Iberomaurusian. After HS 1, however, this region, like the Iberian regions, evidences a simultaneous peak phase in the radiocarbon record which is matched by site numbers and an expansion of settlement areas. Modern ombrotype distributions show more semiarid and dry conditions than Southern Iberia, but the palaeoclimate data record is very sparse and, so far, does not support the notion of a more critical environmental situation in Morocco. An unmodified transference of HS 1 climate simulation from Southern Iberia to Northern Morocco would probably result in even stronger aridity-indexes, but further research is required to clarify this topic.

Compared to the Early Iberomaurusian and the Magdalenian, a marked difference in human adaptation during the Late Iberomaurusian can be identified. After HS1, groups in Morocco heavily exploited land snails as a food resource. Although this has so far only been studied in detail for the Neolithic [114], exploitation begins in the Late Iberomaurusian, after HS 1. The huge number of snail shells in living sites form thick occupation layers, sometimes towering several meters. This is not reported from Southern Iberia, although land snails are abundant there as well. Integrating land snails into the diet could have been the Late Iberomaurusian groups answer to difficult environmental conditions, which need to be addressed in future research.

## Conclusion

Our data from the Westernmost Mediterranean indicates significant changes in settlement patterns and site density after the LGM on both sides of the Strait of Gibraltar. During the Solutrean, major parts of Iberia, with the exception of the Interior and some coastal areas, are consistently populated by hunter-gatherer groups. This pattern abruptly changes with HS 1, as hyperarid zones drastically expand throughout Southern Iberia, from the south-eastern coast via the Interior to the West. High resolution climate modelling indicates that this aridification process is especially severe due to a precipitation minimum at the beginning of the growing season. Magdalenian settlement in the South is probably heavily affected by this climatic impact. The mainly coastally orientated site distribution makes settlement systems and mating networks highly vulnerable to dry spells. During HS 1, networks were probably interrupted by arid zones for several hundreds of years or even shorter periods, causing a strong decline in hunter-gatherer population. Southern Iberia turns out to be a high-risk environment during HS 1 when compared to Northern Iberia, where climate change is less severe and even a contemporaneous expansion of settlement areas is evident. Interestingly, after HS 1 a recovery of the Magdalenian population, marked by site numbers and distribution, is not discernible.

Southern Iberia and Northern Morocco show similar current climate regimes and are thus assumed to be high-risk environments, at least during HS 1. However, the development of human demography is different in both areas during the LGM and in the Late Glacial. In the LGM, with a stable habitation of Southern Iberia, Morocco is weakly occupied. During HS 1, both regions are affected. However, the decrease of human presence as seen in Southern Iberia after the Solutrean is not visible in Morocco, where evidences of the Early Iberomaurusian still slightly expand. After HS 1, the radiocarbon record displays an increase of data, but the overall number of sites in Southern Iberia is still significantly lower when compared to the North and single-layered sites are more abundant. At the same time, we see a constant increase of sites in Morocco during the Late Iberomaurusian. If our site data and the conclusions derived are correct, these differences could be explained by three alternative scenarios:

- Climate differences between regions are stronger or more complex than our data suggest
- Differences in the history of human demography of the two areas are responsible
- Human adaptation to cope with climate stress was different in the two areas

At the moment, it is still difficult to decide which scenario is most likely; further research is needed. However, this study illustrates the high potential of the Westernmost Mediterranean for modelling Late Pleistocene hunter-gatherer behaviour in relation to climate change. The data presented shows changes in settlement patterns in the three macro areas of the Westernmost Mediterranean through time, following clearly different trends. The impact of HS 1 was probably a major driving factor behind changes in the settlement system. While Northern Iberia remains stable and evidences even an expansion of human population, Southern Iberia and Morocco can both be classified as temporary high-risk environments that required special adaptive capacity and adjustment.

## Supporting information

S1 Table Site database of Solutrean and Magdalenian occupations on the Iberian Peninsula and of Iberomaurusian occupations in Morocco compiled for this study. The region, site type, geographical coordinates and presence or absence (–) of archaeological complexes are noted for each site, as is the presence of single (s), multi (m) and unknown (?) stratigraphical subunits for each complex. The ID number corresponds to the labels of Fig 1.
(XLSX)

S2 Table 14C-data. Published radiocarbon dates (uncalibrated) for Late Glacial archaeological sites from Iberia and Morocco (n = 542). IBM = Iberomaurusian; SOL = Solutrean; MAG = Magdalenian.
(XLSX)
